# Impact of Metal-Functionalized Fullerenes on the Proliferation of Pathogenic Fungi

**DOI:** 10.1021/acsomega.5c07052

**Published:** 2026-02-02

**Authors:** Abed Alqader Ibrahim, Tariq Khan, Dennis LaJeunesse, Sherine O. Obare, Anthony L. Dellinger

**Affiliations:** † Department of Nanoscience, Joint School of Nanoscience and Nanoengineering, 14616 University of North Carolina at Greensboro, Greensboro, North Carolina 27402, United States; ‡ Kepley Biosystems Incorporated, Greensboro, North Carolina 27214, United States; § AT Research Partners, Burlington, North Carolina 27217, United States

## Abstract

Given the trajectory and prevalence of multidrug-resistant (MDR) organisms like *Candida auris*, the dearth of available antifungal drugs and the global need for effective therapeutics, the exploration of safe antifungals with broad-spectrum potential and novel antimicrobial mechanisms is imperative for future treatment strategies. Herein, the broad-spectrum potential of previously synthesized silver and copper coordinated chlorine functionalized fullerene nanoparticles (Ag–C_60_–Cl and Cu–C_60_–Cl) against two clinically significant fungal pathogens, *Candida albicans* and *C. auris* is investigated. The experimental results show enhanced antifungal activity of Ag–C_60_–Cl compared to Cu–C_60_–Cl, C_60_–Cl, and fluconazole. The minimum inhibitory concentrations (MIC) of Ag–C_60_–Cl and Cu–C_60_–Cl are 15.62 and 250 μg/mL, respectively, against *C. albicans*. Notably, the MIC of the Ag–C_60_–Cl against *C. auris* is 3.9 μg/mL, whereas the MIC of Cu–C_60_–Cl is 250 μg/mL. Analysis of fungal growth kinetics shows that Ag–C_60_–Cl significantly delayed the growth of *C. albicans* and suppressed the growth of *C. auris*. Mechanistic studies highlight that Ag–C_60_–Cl produced higher reactive oxygen species (ROS) and triggered catalase enzymes by acting as oxidants. Additionally, the NPs exhibited physical interactions with yeast cells, indicating a dual mode of action. These findings establish the potential of Ag–C_60_–Cl as a new and potentially transformative antifungal strategy against two clinically significant pathogens.

## Graphical abstract

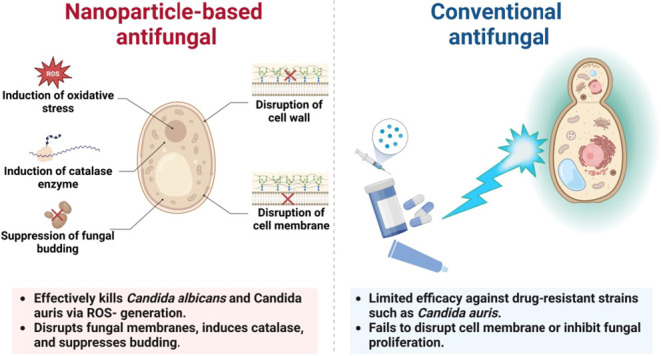

## Introduction

Fungal infections are especially challenging to detect,^1^ and in the absence of early and effective therapy can be difficult to treat. Fungal infections affect over 1 billion people annually, causing 1.6 million deaths worldwide.^2^ These infections impose significant burden on healthcare systems by causing a wide range of health complications, particularly in immunocompromised patients. In recent years, resistance to antifungal treatments has increased substantially, signaling a silent pandemic that poses an emerging global health concern.^3^ In both high-income and resource-limited settings, the trajectory of drug-resistant fungal pathogens represents a cause for concern and presents challenges globally.

Annually, some 30 million people worldwide are diagnosed with sepsis, ranking it as the third leading cause of death overall, outpacing prostate, breast cancer and HIV/AIDS combined, with a 25–30% mortality rate.^4–7^ About one-third of septic patients enter the health system via the emergency department, representing an enormous hospital liability.^8^ Sepsis is the leading cost of hospitalization, at 24 billion USD annually, and the primary cause of US hospital readmissions, with 19% of sepsis patients rehospitalized within 30 days.^4,9,10^ Sepsis cases have steadily increased by 1.5% per year, and related costs have increased nearly 20% since 2011.^9,11^ While the majority of sepsis cases are associated with bacterial pathogens, estimates implicate fungal species in approximately 20%.^12^ Notably, these often contribute to greater morbidity and mortality, between 40 and 60%.^13^ Given that the causative agent of infection is fungal, the administered broad-spectrum antibacterial therapy offers no amelioration or therapeutic benefit to the patient: in fact, it has been shown that broad-spectrum therapies often worsen symptoms.^14^ Kumar et al. previously reported that a 12% decrease in patient survival was observed every hour that appropriate therapy was delayed.^15^

Invasive fungal infections have a high mortality rate (50%), with Candida species (Candida spp.) accounting for nearly 50–60% of all fungal-related deaths.^16^ In clinical settings, certain risk factors, including antibiotic therapy, organ transplantation, neutropenia and prolonged intensive care unit (ICU) stays, increase the prevalence and severity of candidiasis.^17^ More than 200 species of *Candida* have been identified, with more than 40 linked to human infections.^18^ Among these species, *C. albicans* is the most significant eukaryotic pathogens and ranks as the fourth most common cause of blood infections in humans.^19^ Despite these concerns, only four classes of antifungal agents (i.e., azoles, echinocandins, polyenes and allylamines) are available to healthcare practitioners.^20^ When treating refractory fungal disorders, azoles such as fluconazole, itraconazole, voriconazole and posaconazole are considered front-line treatments.^21^ Due to the rapid development of drug resistance in fungi, current antifungal agents are becoming increasingly ineffective, especially when compared to antibiotics, which have more than 10 distinct classes.^22^ For instance, *C. auris* is an emergent and highly virulent fungal pathogen that poses a significant global health threat due to its multidrug resistance, high mortality rates, as well as its tendency to spread rapidly in hospital and clinical facilities.^23^

Healthcare systems require innovative strategies that advance the efficacy and treatment of fungal infections. Offering novel mechanisms of action that can overcome resistance, nanoparticles (NPs) have gained traction as effective antimicrobial agents due to their unique physiochemical properties, including their high surface area-to-volume ratios, customizable surface functionalities, small size, controllable surface charge, shape, electron transfer, and redox-active properties.^24^ These properties can be tailored to enhance biocompatibility and therapeutic efficacy. The ability of NPs to overcome limitations associated with traditional antifungal therapies (i.e., drug resistance, limited spectrum of activity, and safety concerns) has positioned these agents as a future solution for combating pathogens.

One prominent class of nanomaterials, metal-based NPs such as silver (Ag), gold (Au) and platinum (Pt) have demonstrated promising antimicrobial properties.^25^ Monteiro et al. described the ability of AgNPs to disrupt biofilms of *C. albicans* and *Candida glabrata* (*C. glabrata*) by targeting the cell wall, suppressing hyphal development and reducing the release of an extracellular polymeric substance (EPS).^26^ In a separate study by Monteiro et al., *Candida* biofilm treatment with AgNPs caused defects in membrane permeability and resulted in the release of intracellular contents, inhibition of respiratory chain enzymes, and prevention of replication.^27^

In this study, previously characterized silver-coordinated chloro-fullerenes NPs (Ag–C_60_–Cl) and copper-coordinated chloro-fullerenes NPs (Cu–C_60_–Cl) synthesized through a rapid one-step reaction that can accommodate up to six silver or copper ions were examined for activity against two clinically relevant fungal species, *C. auris* and *C. albicans*.

## Experimental Section

### Materials

Fullerenes (C_60_; 99.95+%) were obtained from SES Research (Houston, TX, USA). Chloroform (anhydrous, ≥99%), silver nitrate (AgNO_3_; ACS reagent, ≥99.0%) and copper­(II) nitrate hydrate (Cu­(NO_3_)_2_; ≥99.9% trace metals basis) were purchased from Sigma-Aldrich Corporation (St. Louis, MO, USA). Fluconazole was purchased from Supelco (Pharmaceutical secondary standard).

### Nanoparticles Synthesis

The Ag–C_60_–Cl and Cu–C_60_–Cl NPs were synthesized and characterized using a rapid one-step reaction as previously published and described in Supporting Information.^28^ Briefly, C_60_ fullerenes (5 mg) were dissolved in chloroform (5 mL) and subsequently sonicated for 10 min using a bath sonicator. Following dissolution and sonication, 2 mL of the desired salt solution was added dropwise to the C_60_ solution under probe sonication for an additional 10 min. The mixture was centrifuged (10,000 rpm, 10 min each) and washed three times with deionized water to remove residual reactants. The resultant nanoparticles were then dried in the air for further evaluation and characterization, as previously published and provided in the Supporting Information.

### Characterization

Fourier-transform infrared spectroscopy (FTIR) spectra were obtained using an Agilent 670 FTIR Spectrometer w/ATR to confirm the molecular functionalization. Samples were analyzed directly, with air used as the blank. To further evaluate the colloidal stability and surface charge characteristics of the nanoparticles, the particle size, distribution, and zeta potential of the nanoparticles were measured using a Malvern ZEN3600 Zetasizer. Dynamic Light Scattering (DLS) measurements were performed in triplicate, and the reported values represent the averaged results.

### Microbial Strains and Culture Conditions

Two infectious fungal species, *C. albicans* (ATCC 90028) and *C. auris* (PR#0385, CDC *C. auris* panel) strains,^29^ were investigated in this study. The *C. auris* strain used for these experiments belongs to phylogenetic lineage, clade IV, and was resistant to treatment with amphotericin B and azoles, as well as showed reduced sensitivity to echinocandins according to the CDC Antimicrobial Resistance Isolate Bank (CDC, 2023).^30^ All microbial strains were stored as glycerol stocks at −80 °C. Yeast cells were cultured in liquid onto agar plates comprised of yeast-peptone-dextrose (YPD) medium and on YPD plates containing 1% agar (wt/vol). All cultures were grown 30 °C in an orbital shaker (120 rpm). The growth medium, yeast-peptone-dextrose (YPD), was purchased from Fisher Scientific (Waltham, MA, USA). The fluorescent probe, H_2_DCFDA (2′,7′-dichlorodihydrofluorescein diacetate), was obtained from Thermofisher, (cat. #D399, Waltham, MA, USA). Amplex Red Hydrogen Peroxide/Peroxidase assay kit (Invitrogen, #A22188) was purchased from ThermoFisher Scientific (Waltham, MA, USA). Human blood for the hemolysis assay was collected from healthy donors in K2-EDTA vacutainers (Innovative Research, Novi, MI, USA).

### Disc Diffusion Assay for Antifungal Activity

The susceptibility of *C. albicans* and *C. auris* to Ag–C_60_–Cl and Cu–C_60_–Cl NPs was determined using a disc diffusion assay. The method was performed in 90 mm Petri dishes comprising YPD agar. First, plates were inoculated by swabbing the agar with a swab containing a yeast suspension of 1 × 10^6^ to 2.5 × 10^6^ cells/mL. A sterile Whatman No. 1 paper was cut into discs of 6 mm in diameter, sterilized by autoclaving for 15 min at 70 °C and loaded with 10 μL (1000 μg/mL) of each of the NPs. The discs were placed on *C. albicans* and *C. auris* inoculated plates. Similarly, the reference standard (fluconazole) and nonfunctionalized NP control (C_60_–Cl) were evaluated to compare the efficacy of the functionalization to the front-line azole antifungal agent and nonmetallic functionalized fullerenes. Fluconazole has an established antifungal activity and provides a reliable benchmark for comparing and evaluating the efficacy of the functionalized NPs used in this study. The zones of inhibition were monitored during incubation and the diameter for each treatment in both species was measured according to CLSI guidelines after incubation.^31^ The presence or absence of a growth inhibition halo around the samples was observed to assess qualitative antimicrobial efficacy.

### Minimum Inhibition Concentration of Nanoparticles

A standard microdilution assay was used to determine the MIC of C_60_–Cl, Ag–C_60_–Cl, and Cu–C_60_–Cl NPs and fluconazole. 2-fold serial dilutions of NPs or the reference standard, ranging from 250 μg/mL to 0.4883 μg/mL, were prepared in YPD broth containing an adjusted fungal concentration equivalent to 0.5 McFarland standard in a 96-well plate. As an internal control, to assess the baseline fungal growth with NPs or fluconazole, the inoculated broth alone was incubated for 24 h at 30 °C. The OD_630_ of each well was measured on a microplate reader (BioTek Synergy H1Multimode Reader) at 0 and 24 h.^32^ The MIC was defined as the lowest concentration of NPs that resulted in inhibition in growth in comparison to the control wells. To ensure accuracy, MIC values were validated by analyzing the absorbance values and visual turbidity of each well before and after 24 h of incubation.

### Growth Kinetics Study

The effect of NPs on the growth kinetics of *C. albicans* and *C. auris* was determined using a microplate reader (BioTek Instruments, Inc.). Briefly, an equal volume of the adjusted inoculum (0.5 McFarland Standard) for each fungal isolate was added to the YPD growth medium in a flat-bottom 96-well microtiter plate. Nanoparticles or the reference standard were introduced at their respective MIC concentrations to evaluate growth inhibition. Immediately following inoculation and treatment, fungal growth kinetics were monitored over 36 h at 30 °C, with OD_630_ measurements taken at 30 min intervals. The resulting growth curves were analyzed to determine the following kinetic parameters: lag phase duration (time required to initiate exponential growth), maximum value (highest absorbance measurement), time required to reach the maximum value and average growth rate (average increase in sample absorbance from 0 to 36 h).

### Quantification of Reactive Oxygen Species

ROS production was measured using dichloro-dihydro-fluorescein diacetate (H_2_DCFDA) following the protocol described in Pérez et al. protocol.^33^ *C. albicans* and *C. auris* were cultured in a YPD medium until OD_630_ reached 0.5. The cells were then harvested by centrifugation, washed with 10 mM potassium phosphate buffer (pH 7.0) and disrupted by sonication after resuspension in the same buffer. The oxidant-sensitive probe, H_2_DCFDA, was dissolved in dimethyl sulfoxide (DMSO), and the ROS-sensitive probe was added to the cell suspension at a ratio of 1:2,000. Following loading of the probe, the samples were incubated with shaking at 30 °C for 30 min. The excess H_2_DCFDA was removed via centrifugation, the resulting cell pellet was washed twice in 1× PBS, and the probe-loaded cells were resuspended in the fresh buffer. Both fungal strains were incubated with either 0.5× MIC of Ag–C_60_–Cl, Cu–C_60_–Cl and C_60_–Cl NPs or fluconazole at the determined MIC to compare the oxidative stress induction induced by the NPs. Untreated fungal cells in media alone served as negative controls for baseline ROS production and hydrogen peroxide (H_2_O_2_; 1 mM) was used as a positive control to confirm elevated ROS levels. After the treatment period, the fluorescence intensity of the oxidized probe, 2′,7′-dichlorofluorescein (DCF), was measured in a fluorescence spectrophotometer/imager (BioTek Cytation 5 Cell Imaging Multimode Reader) at an excitation wavelength of 488 nm and an emission wavelength of 535 nm.

### Measurement of Catalase Activity

To evaluate the catalase activity of the samples, the Amplex Red Catalase Assay Kit (A22180; Invitrogen) was performed according to manufacturer instructions. The Amplex Red reagent stock solution was prepared by dissolving 0.26 mg of the reagent in 100 μL of DMSO (10 mM). A 1× reaction buffer was prepared by combining 4 mL of 5× Reaction Buffer stock with 16 mL of deionized water. Additionally, a 100 U/mL HRP solution and a 20 mM H_2_O_2_ working solution were prepared. In parallel, a 1000 U/mL catalase solution was made by dissolving catalase in 100 μL deionized water. For the assay, samples were diluted in 1× Reaction Buffer and pipetted into a microplate. A 40 μM H_2_O_2_ solution was then added and incubated for 30 min. During incubation, a working solution of 100 μM Amplex Red reagent with 0.4 U/mL HRP was prepared. Next, 50 μL of the Amplex Red/HRP working solution was added to each well-containing samples with 0.5× MIC concentrations of nanoparticles or the reference standard (untreated fungi cells grown under the same conditions), alongside appropriate catalase enzyme controls (no-catalase; negative control and 1000 U/mL catalase enzyme; positive control). The microtiter plate was then incubated for at least 30 min at 30 °C and protected from light. The fluorescence was measured using a microplate reader (BioTek Cytation 5 Cell Imaging Multimode Reader) with excitation in the 530–560 nm range and emission detection at approximately 590 nm for a duration of 4 h at 30 °C inside the plate reader. The change in fluorescence intensity was determined by subtracting the sample value from that of the no-catalase control.

### Evaluation of Nanoparticles-Fungi Interaction

The specific interactions of NPs and fungi were recorded using Scanning Electron Microscopy (SEM) (JEOL JSM-IT800 FESEM with Oxford Ulti Max EDS). Specimens were prepared using a modified fixation protocol based on standard procedures.^34^ A glutaraldehyde-formaldehyde mixture (Karnovsky’s fixative) was used for primary fixation to preserve cellular morphology. The fungi were treated with MIC concentrations of the NPs and incubated for 2 to 4 h. After NP treatment and exposure, specimens were washed three times using phosphate buffer saline (PBS) to remove excess fixative and NPs. All samples were immersed in the fixation solution overnight, followed by three centrifugation and washing steps using 0.1 M cacodylate buffer, pH 7.4. Lastly, a gradual dehydration was performed using ethanol at increasing concentrations: 35%, 50%, 95% and 100%. The samples were delicately centrifuged between each rinsing and dehydration step to ensure proper pelleting of fungal cells using a low-speed centrifugation cycle (4,000 RCF for 5 min at room temperature) to avoid damage to the fungal cells. The SEM stubs were washed with ethanol and subsequently cleaned with plasma. Finally, the fungal suspension was deposited onto a clean Si wafer and all samples were sputter-coated with a 7 nm layer of gold–palladium prior to imaging.

### Determination of Cytotoxicity Effects of Nanoparticles

A hemolysis assay was conducted to evaluate the cytotoxicity of the NPs as described by Wang et al. with slight modifications.^35^ Initially, 2 mL of a blood sample obtained from a healthy donor was centrifuged at 1500 rpm to separate red blood cells (RBCs) from the plasma and the buffy coat. The RBCs were then washed three times with phosphate buffer saline (PBS) to remove any residual plasma. A solution of 5% RBCs was then prepared for further use. Increasing concentrations of the C_60_–Cl, Ag–C_60_–Cl, Cu–C_60_–C, and fluconazole (2.5, 25, 50, and 100 μg/mL) were prepared in 475 μL PBS before the addition of 25 μL of the 5% RBCs. The solutions were then incubated at 37 °C with gentle shaking for 1 h. Intact RBCs were pelleted by centrifugation at 1500 rpm for 10 min and the supernatant was collected. The absorbance of the supernatant was measured at 540 nm using a microplate reader. Deionized water served as the positive control (100% lysis) and PBS was used as the negative control (0% lysis). The hemolysis results were calculated using the equation

$$\mathrm{hemolysis}\;\left(\%\right)=\frac{\left(A_{s}-A_{\mathrm{nc}}\right)}{\left(A_{\mathrm{pc}}-A_{\mathrm{nc}}\right)}$$

*A*_s_ represents the 540 nm absorbance of each sample; *A*_nc_ is the absorbance of the negative control (0% hemolysis of the untreated sample); and *A*_pc_ is the absorbance of the positive control (100% hemolysis of the untreated sample). All experiments were conducted in triplicate.

### Statistical Analysis of Antifungal Activity

Fluconazole was used as a reference antifungal agent to analyze the comparative antifungal efficacy between the synthesized NPs against the two fungal species. Fluconazole was selected as the reference standard because this azole represents a front-line treatment strategy and is a member of the most clinically relevant subgroup exhibiting high antifungal efficacy and low toxicity. All experiments were performed in triplicate and the results are presented as mean values with the standard error of the mean included for statistical accuracy. Statistical analyses were performed using a two-tailed *t* test to assess the significance of differences between the nanoparticle treatments and the reference standard.

## Results and Discussion

### Fourier-Transform Infrared Spectroscopy

The successful functionalization of the nanoparticles and metal coordination with fullerenes was verified by FTIR spectroscopy. Significant variations in vibrational modes have been shown in the spectra of pristine fullerene (C_60_), chloro-fullerene (C_60_–Cl), and metal-coordinated fullerene complexes (Ag–C_60_–Cl and Cu–C_60_–Cl) as shown in Figure 1. The distinctive absorption peaks associated with the vibrational modes of fullerenes are shown in FTIR spectra of C_60_. The primary infrared-active modes, attributed to the highly symmetric structure of C_60_, appear at 526 cm^–1^ (radial breathing mode), 576 cm^–1^ (F_1u_ symmetry), 1182 cm^–1^, and 1427 cm^–1^. The distinct sp2 hybridization and spherical geometry of fullerenes are observed by these peaks.

**Figure 1:**
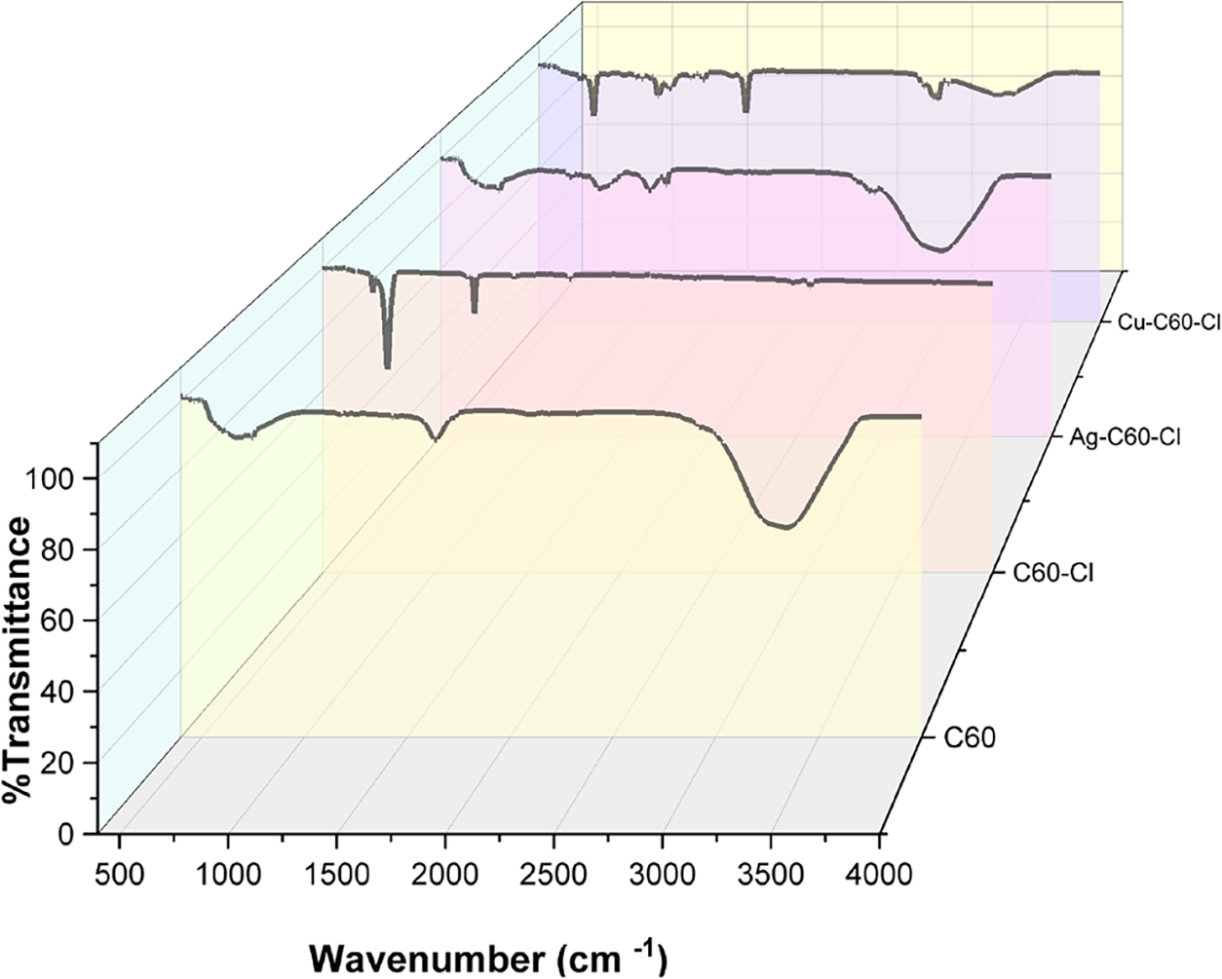
FTIR spectra of C_60_, C_60_–Cl, Ag–C_60_–Cl, and Cu–C_60_–Cl fullerenes.

Significant alterations in the FTIR spectra were observed upon functionalization with chlorine. A sharp new peak appears at 1214 cm^–1^, which can be attributed to the C–Cl stretching vibration, confirming successful functionalization. The peak at 1729 cm^–1^, present in both C_60_ and C_60_–Cl spectra, likely corresponds to a carbonyl (C=O) group, suggesting possible oxidation of the fullerene nanoparticles. Notably, the peak at 1639 cm^–1^ observed in C_60_ disappeared in the C_60_–Cl spectrum, indicating that functionalization alters the vibrational mode associated with this wavenumber. Furthermore, while the peak at 576 cm^–1^, attributed to the radial breathing mode of C_60_, is significantly reduced in C_60_–Cl, the peak at 1427 cm^–1^ remains unchanged. This indicates that the core structure of C_60_ is largely preserved, but the vibrational modes associated with the fullerenes are modified by the chlorination.

Moreover, notable shifts in vibrational frequencies result from the coordination of silver to the chloro-fullerene. A new peak appears around 1340 cm^–1^, representing a modified vibrational mode associated with the interaction between the silver ion and the fullerene structure. Furthermore, the appearance of the peak at 1635 cm^–1^ suggests new vibrational interactions within the fullerene, due to the influence of silver on the π-conjugated system of the fullerene, specifically the C=C bonds. These shifts and new peaks provide evidence that the coordination is likely occurring through the fullerene’s double bonds, a common interaction observed in fullerene metal systems, further supported by minor shifts in peak associated with the fullerene core structure. In addition, the peak corresponding to the carbonyl stretch shifts slightly from 1729 cm^–1^ to 1733 cm^–1^, which suggests an interaction between the carbonyl group and the silver ion. This shift, alongside the increased sharpness of the peak, indicates potential stabilization of the C=O bond due to metal coordination.

The Cu–C_60_–Cl spectrum similarly shows a shift in the carbonyl peak to 1735 cm^–1^. However, this peak appears sharper compared to the Ag–C_60_–Cl spectrum. The sharpness indicates a stronger interaction between the copper ion and the fullerene carbonyl group, which could be due to the higher Lewis acidity of copper compared to silver. Additionally, there are small shifts in the C–Cl and fullerene-related peaks, signifying that copper has a more pronounced effect on the overall structure compared to silver.

### Dynamic Light Scattering Analysis

DLS measurements were performed in triplicates, and the averaged values were reported. The Ag–C_60_–Cl NPs exhibited a mean hydrodynamic diameter of 827.31 ± 63.32 nm with a polydispersity index (PDI) of 0.572 ± 0.037, indicating a moderately uniform particle distribution. The corresponding zeta potential was −47.48 ± 1.25 mV, suggesting strong electrostatic repulsion between particles and excellent colloidal stability in suspension. Cu–C_60_–Cl NPs displayed a larger hydrodynamic diameter of 1044.48 ± 56.99 nm and a higher PDI of 0.941 ± 0.054, implying a broader size distribution. The zeta potential was +25.40 ± 2.02 mV, indicating moderate colloidal stability. The control C_60_–Cl NPs showed a hydrodynamic size of 774.95 ± 145.11 nm with a PDI of 0.847 ± 0.107, and a zeta potential of −58.38 ± 1.74 mV, reflecting superior colloidal stability. This strong negative surface potential supports sustained dispersion, enhancing bioavailability and consistent interaction with microbial targets.

### Antifungal Activity of Nanoparticles

The antifungal activity of C_60_–Cl, Ag–C_60_–Cl, Cu–C_60_–Cl and fluconazole against two Candida species (Candida spp.) was determined by zone of inhibition as shown in Figure 2. The nonmetallic NPs (C_60_–Cl) revealed no detectable zone of inhibition against either *C. albicans* or *C. auris* (Figure 2; top right quadrants). In contrast, Ag–C_60_–Cl exhibited significant antifungal activity against both *C. albicans* (16.2 ± 0.4 mm) and *C. auris* (19.4 ± 0.5 mm) (Figure 2; bottom right quadrants). Cu–C_60_–Cl demonstrated greater antifungal activity compared to nonmetallic NPs but was less profound compared to Ag–C_60_–Cl in both fungal species. Of all treatments, fluconazole exhibited the greatest activity against *C. albicans* (27.2 ± 1.4 mm) when compared to all other treatments, there was no activity observed against *C. auris*, which has been shown to be resistant to this drug.^29^ These findings demonstrated that Ag–C_60_–Cl exhibited greater antifungal activity compared to both nonmetallic NPs and Cu–C_60_–Cl in both fungal species. Furthermore, while fluconazole showed the greatest activity among all treatments against *C. albicans*, no antifungal activity was observed against *C. auris*, whereas the antifungal activity of the Ag–C_60_–Cl was maintained.

**Figure 2:**
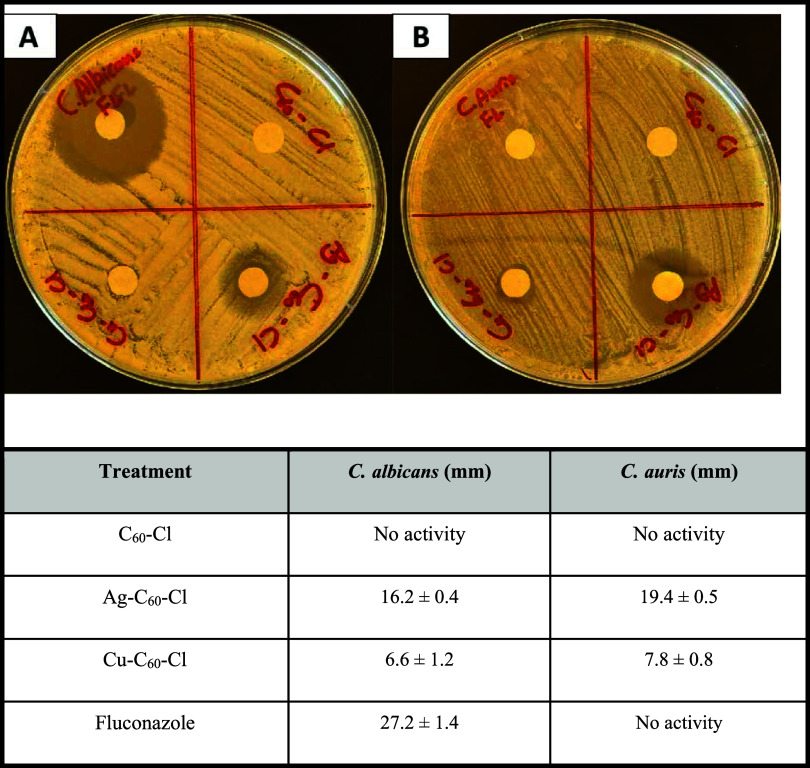
Representative agar plates demonstrating the antifungal activity of C_60_–Cl (nonmetallic NPs), Ag–C_60_–Cl, Cu–C_60_–Cl and fluconazole against (A) *C. albicans* and (B) *C. auris*. The zone of inhibition was determined by measuring the full diameter of the clear zone using a transparent ruler. Values represent the mean ± standard deviation for five identical replicates.

### Minimum Inhibition Concentration of Nanoparticles

A microdilution assay was performed to obtain the MIC values of each NP treatment and fluconazole, as shown in Table 1. Absorbance and turbidity measurements were recorded at different concentrations (0.48825, 0.9765, 1.953, 3.906, 7.8125, 15.625, 31.25, 62.5, 125.0, and 250.0 μg/mL) after 24 h. The results showed that the nonmetallic fullerenes (C_60_–Cl) showed no antifungal activity at concentrations up to 250 μg/mL for either strain. Whereas the Ag–C_60_–Cl demonstrated strong antifungal activity, with MIC values of 15.625 μg/mL against *C. albicans* and 3.906 μg/mL against *C. auris*. On the other hand, Cu–C_60_–Cl showed moderate activity with an MIC of 250 μg/mL against both *Candida* species. The front-line azole (fluconazole) showed strong antifungal activity against *C. albicans* (MIC = 3.906 μg/mL) but was ineffective against *C. auris* (MIC > 250 μg/mL).

**Table 1:** Minimum Inhibitory Concentration (MIC) Values (μg/mL) for C_60_–Cl, Ag–C_60_–Cl, Cu–C_60_–Cl, and Fluconazole against Two Candida spp., *C. albicans* and *C. auris*

	MIC (μg/mL)			
strain	C_60_–Cl	Ag–C_60_–Cl	Cu–C_60_–Cl	fluconazole
*C. albicans*	>250	15.625	250	3.906
*C. auris*	>250	3.906	250	>250

### Time-Dependent Differential Growth Inhibition

The fungicidal activity of the NPs was further evaluated by investigating the growth curves of the two Candida spp. The rate and extent of growth inhibition were determined by observing and measuring the optical density over a period of 36 h. The growth inhibition of the yeasts by fluconazole, C_60_–Cl, Ag–C_60_–Cl and Cu–C_60_–Cl NPs, as well as nontreated control samples recorded as a function of time, suggested significant differences in antifungal activity between each treatment, as shown in Figure 3. In *C. albicans* (Figure 3a), Cu–C_60_–Cl and C_60_–Cl revealed no growth inhibition compared to the untreated control, beginning exponential growth at approximately 9 h. However, treatment with Ag–C_60_–Cl revealed potential fungicidal activity in *C. albicans* causing a delay in the growth and shifting the exponential phase to around 19 h as shown in Figure 3a and Table 2. This reduction or impairment of early proliferation and subsequent extension of the *C. albicans* lag phase indicates potential antifungal activity, likely exerting inhibitory effects on key metabolic or reproductive process, inducing stress, or some combination thereof. As previously described in the literature, fluconazole significantly suppressed the growth of *C. albicans* which corroborated the observed disc diffusion (Figure 2) and MIC results (Table 2). Conversely, fluconazole treatment revealed partial inhibitory effects on the growth curve of *C. auris* (Figure 3b), which correlated with previous reports and the MDR phenotype. In similar agreement with the disc diffusion (Figure 2) and MIC (Table 2) data, Cu–C_60_–Cl and C_60_–Cl pretreatment had minimal impact on the growth kinetics of *C. auris* when compared to the untreated control. Most notably, pretreatment of *C. auris* with Ag–C_60_–Cl revealed the greatest fungicidal activity among all treatments, significantly inhibiting the growth pattern, extending the lag phase, and revealing no growth until around 29 h, which was comparable to the behavior of fluconazole in *C. albicans*.

**Figure 3:**
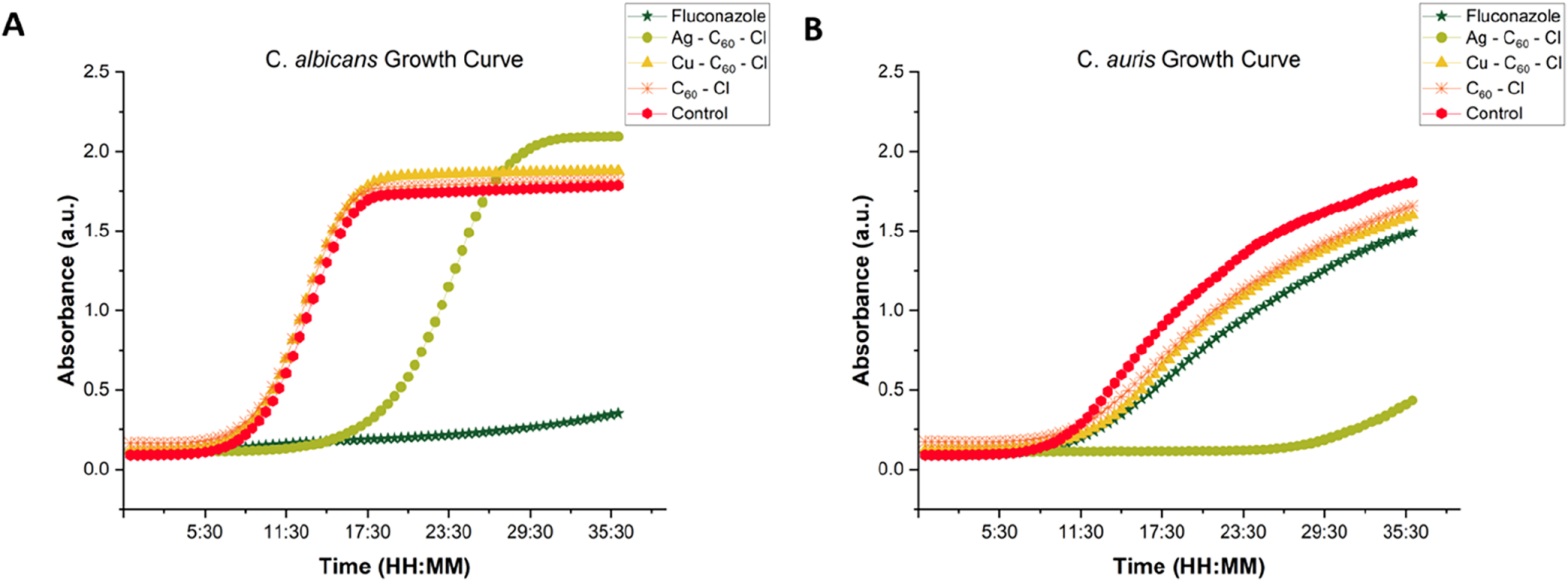
36 h growth of (A) *C. albicans* (B) *C. auris* in the presence of fluconazole, Ag–C_60_–Cl, Cu–C_60_–Cl, and C_60_–Cl NPs at their respective MIC.

**Table 2:** Kinetic Growth Parameters of *C. albicans* and *C. auris* under Different Treatments

	lag phase (HH/MM)		maximum value (a.u.)		time at max value (HH/MM)		avg. growth rate (a.u./h)	
condition	*C. albicans*	*C. auris*	*C. albicans*	*C. auris*	*C. albicans*	*C. auris*	*C. albicans*	*C. auris*
fluconazole (solid green star)	20:11 ± 01:09	11:16 ± 01:10	0.35 ± 0.08	1.50 ± 0.21	34:40 ± 00:34	18:00 ± 01:48	0.01 ± 0.002	0.04 ± 0.000
Ag–C_60_–Cl (light green circle solid)	19:01 ± 00:34	29:08 ± 01:24	2.09 ± 0.05	0.43 ± 0.50	24:00 ± 00:51	35:00 ± 00:00	0.06 ± 0.001	0.01 ± 0.005
Cu–C_60_–Cl (yellow triangle up solid)	09:12 ± 00:04	11:59 ± 00:32	1.88 ± 0.02	1.60 ± 0.24	12:40 ± 00:17	17:10 ± 00:45	0.05 ± 0.000	0.04 ± 0.001
C_60_–Cl (orange punctuation mark)	09:19 ± 00:04	11:14 ± 00:50	1.82 ± 0.04	1.66 ± 0.22	12:50 ± 00:17	17:20 ± 01:36	0.05 ± 0.000	0.04 ± 0.000
control (red hexagon solid)	09:25 ± 00:10	11:17 ± 00:25	1.79 ± 0.02	1.81 ± 0.29	13:00 ± 00:00	14:20 ± 01:09	0.05 ± 0.000	0.05 ± 0.002

### Reactive Oxygen Species Quantification

Upon exposure to stressors, like fungicidal agents, Candida spp. are known to generate ROS as part of their cellular response and potentially leading to oxidative damage within the fungal cells. Previous research has shown that metallic nanoparticles, upon interaction with microbial cells, can induce the production of ROS, which in turn plays an important role in their antimicrobial action.^36^ This induced oxidative stress can cause damage to vital cellular components, including membrane lipids, proteins and DNA.^37^ Furthermore, fullerenes themselves have also been reported to generate or scavenge ROS depending on structural modifications and environmental conditions, illustrating the duality of these particles.^28^ To investigate the role of oxidative stress in antifungal activity, ROS generation in *C. albicans* and *C. auris* was quantified following exposure to C_60_–Cl, Ag–C_60_–Cl and Cu–C_60_–Cl. ROS levels were assessed using H_2_DCFDA fluorescence dye and presented as relative fluorescence intensity with untreated controls, hydrogen peroxide (H_2_O_2_), and the front-line azole (fluconazole) included for comparison (Figure 4).

**Figure 4:**
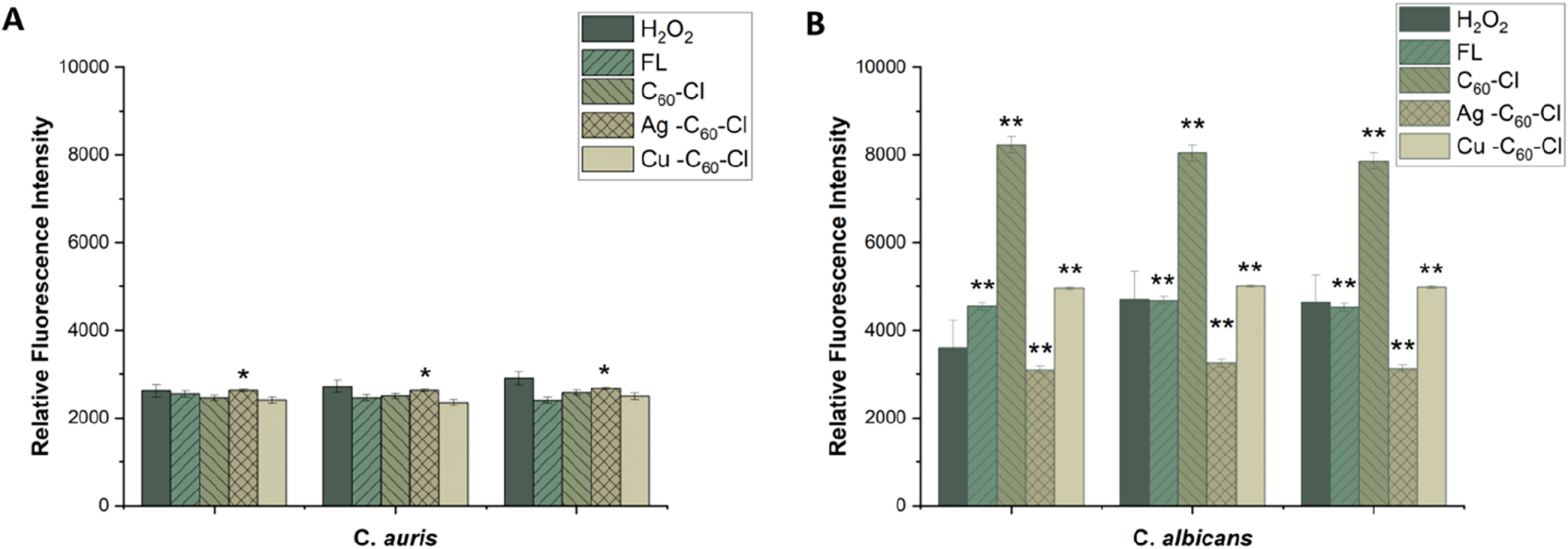
Relative fluorescence intensity indicating ROS production in *C. albicans* and *C. auris* treated with fluconazole, C_60_–Cl, Ag–C_60_–Cl and Cu–C_60_–Cl fullerenes. (a) *C. albicans* and (b) *C. auris* were treated with 0.5× MIC of fluconazole, C_60_–Cl, Ag–C_60_–Cl, and Cu–C_60_–Cl fullerenes, or H_2_O_2_ control. Data is represented by three independent assays (±SD). Statistical analysis using a two-tailed *t* test was performed to assess the significance of differences between the nanoparticle treatments. Significance is denoted as *p* < 0.05 (*) and highly significant *p* < 0.005 (**).

The data revealed that the different treatments resulted in different levels of ROS production. Specifically, *C. albicans* revealed higher overall ROS levels compared to *C. auris*. In *C. albicans*, treatment with the nonmetallic fullerene (C_60_–Cl) at 0.5× MIC (125 μg/mL) induced a statistically significant increase in ROS generation (*p* < 0.01) that was nearly 2-fold greater than H_2_O_2_, a well-established benchmark for measuring oxidative stress. These results paralleled our previously published results with C_60_–Cl causing the highest ROS production in *E. coli*. However, the confocal images (Figure 5) suggest that this increase in oxidative stress is an artifact attributed to the presence of high background fluorescence and increased cell counts (as C_60_–Cl revealed no antifungal activity to Candida spp.; Figure 2 and Table 1). Notably, the most potent fungicidal NP, Ag–C_60_–Cl (MIC = 15.625 μg/mL) showed the lowest ROS production, proposing that the observed antifungal action takes place through a different mechanism. However, this low ROS production is significant in comparison to the ROS production by other treatments due to concentration differences, wherein the 0.5× MIC of Ag–C_60_–Cl against *C. albicans* was 7.8 μg/mL and the rest were 125 μg/mL.

**Figure 5:**
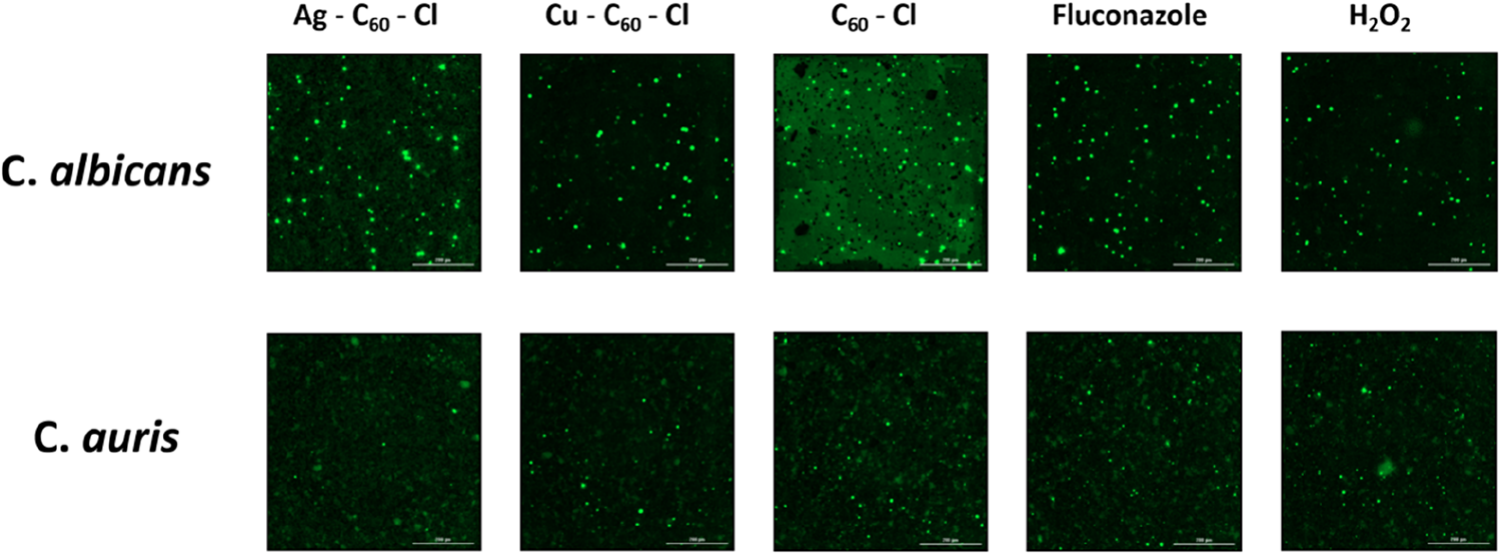
Confocal scanning fluorescence images of *C. albicans* (upper row) and *C. auris* (lower row) treated with fluconazole, C_60_–Cl, Ag–C_60_–Cl, Cu–C_60_–Cl, H_2_O_2_, and untreated control.

Conversely, *C. auris*, upon treatment with all types of fullerenes, revealed generally lower ROS production than those observed in *C. albicans*. In contradiction to *C. albicans*, the *C. auris* ROS results revealed the highest ROS production upon exposure to Ag–C_60_–Cl NPs, potentially differentiating the mechanism of action in *C. auris*. It has been studied that various bacteria capable of tolerating and neutralizing ROS will produce less fluorescence intensity. This may further be a function of efflux pump activity that can rapidly expel the ROS-detecting fluorescent probe from the cells and impair accurate analysis of the results. This behavior is well explained and matches the literature as *C. auris* has a unique characteristic that distinguishes it from other species. In *C. auris*, the upregulation of some genes encoding efflux pumps, including major facilitator superfamily (MFS) transporters and ATP-binding cassette (ABC), contributes to *C. auris*’ resistance to antifungal drugs. Considering the possible effects associated with efflux pumps, higher intrinsic antioxidant enzyme activities, such as catalase could contribute to lower net ROS levels (Figure 6).

**Figure 6:**
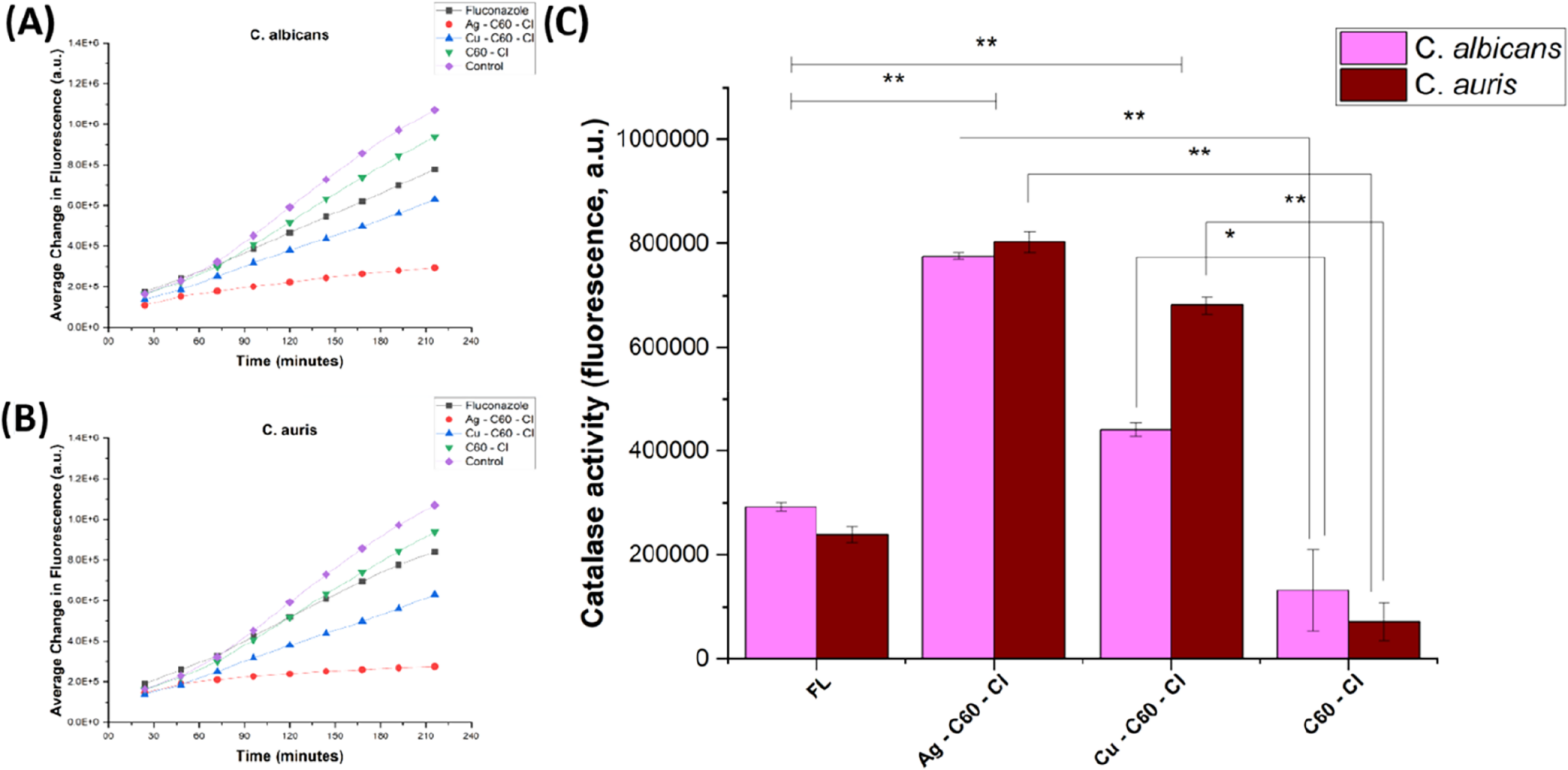
Time-dependent change in fluorescence in (A) *C. albicans* and (B) *C. auris* treated with fluconazole, C_60_–Cl, Ag–C_60_–Cl, Cu–C_60_–Cl nanoparticles, and untreated control. (C) Change in fluorescence indicating catalase activity in *C. albicans* and *C. auris* treated with C_60_–Cl, Ag–C_60_–Cl, and Cu–C_60_–Cl nanoparticles, and untreated control. Both *C. albicans* and *C. auris* were treated with fluconazole, C_60_–Cl, Ag–C_60_–Cl, and Cu–C_60_–Cl fullerenes with 0.5× MIC concentrations, along with appropriate controls. Data is represented by three independent assays (±SD). Significance is defined as *p* < 0.05 (*) and highly significant *p* < 0.005 (**).

As described in previous work, Ag–C_60_–Cl NPs were used at a low concentration of 0.5× MIC (1.953 μg/mL against *C. auris*), significantly lower than the 125 μg/mL of C_60_–Cl (nonmetallic NP). Despite this, Ag–C_60_–Cl demonstrated a comparable ROS fluorescence signal and fewer viable cells (Figure 5). These results suggest that the coordination of silver on the fullerenes enhances the activity of the NPs, stimulating similar ROS generation at a 128-fold lower concentration. The results further revealed that Cu–C_60_–Cl was able to generate higher levels of ROS against *C. albicans* but was observed to be markedly lower against *C. auris*. This differential response may be attributed to the role of copper as a ROS scavenger. It is further hypothesized that the ROS generated by exposure to C_60_–Cl could have been subsequently quenched by the associated copper present in the Cu–C_60_–Cl NPs.

A two-tailed *t* test statistical analysis was performed to assess the significance of the differences between the NP treatments. In *C. albicans*, a significant difference (*p* < 0.05) between both Ag–C_60_–Cl, Cu–C_60_–Cl and the nonmetallic C_60_–Cl NPs indicating a remarkable change in the response upon the addition of the metals. In a comparison between the NPs and the fluconazole, all *t* test values resulted in a significant difference (*p* < 0.05) indicating a major difference in the mechanism of action for the NPs and the fluconazole in the *C. albicans*.

In *C. auris*, a significant difference (*p* < 0.05) between Ag–C_60_–Cl and C_60_–Cl NPs was observed, indicating that the silver functionalization has a major enhancement on the ROS generation compared to C_60_–Cl NPs. However, the insignificant difference between Cu–C_60_–Cl and C_60_–Cl NPs could be attributed to the previously mentioned copper role in scavenging ROS. The interesting comparison between the NPs and fluconazole showed only a significant difference between Ag–C_60_–Cl and fluconazole (*p* < 0.05) suggesting that Ag–C_60_–Cl NPs were able to stress C. *auris* cells when other treatments showed almost no effect.

### Catalase Activity

Catalase is an antioxidant enzyme that can be triggered downstream in response to oxidative damage to fungi cells. As proposed above, ROS generation caused by NP exposure may induce higher catalase production, which acts as its antioxidant defense system.^38^ In this study, we evaluated the time-dependent response to oxidative stress induced by fluconazole, C_60_–Cl, Ag–C_60_–Cl and Cu–C_60_–Cl NPs in both species, *C. albicans* and *C. auris* (Figure 5 A,B). C_60_–Cl shows the highest change in fluorescence with time, which indicates the lowest catalase activity induced in both *C. albicans* and *C. auris*. Based on the ROS data from the C_60_–Cl experiment, we expected that the catalase activity would have been higher, however, we observed that the C_60_–Cl has low oxidative stress on the fungi cells and the previous ROS results confirm that it is attributed to the observed high background fluorescence for C_60_–Cl in the ROS confocal images (Figure 5). This artifact resulted in an overestimation of oxidative stress, which was subsequently corroborated by catalase results (Figure 6).

Fluconazole is an antifungal drug that targets ergosterol synthesis by inhibiting the enzyme lanosterol 14-α-demethylase rather than inducing significant ROS generation. Consistent with the mechanism of action, some mild oxidative stress can be observed after fluconazole treatment, with minimal increase in catalase activity (Figure 6). Copper is an essential micronutrient for fungi, driving many essential biochemical processes.^39^ However, excess copper is toxic resulting in ROS generation via the Fenton reaction, causing damage to cellular membranes, nucleic acids, and proteins.^39^ Candida spp. have evolved specific defense mechanisms that mitigate copper toxicity. Cu–C_60_–Cl NPs induced greater catalase activity than fluconazole (Figure 6), however treatment did not exhibit fungicidal activity (Figures 2 and 3 and Table 1), suggesting that fungi effectively manage this stress through intrinsic copper homeostasis pathways. In contrast, the inclusion of silver in the modified Ag–C_60_–Cl NPs, which exerts additional cytotoxic effects (compared to copper) induced the highest catalase activity (Figure 6). It can be inferred from the disc diffusion (Figure 2), growth curves (Figure 3) and MIC (Table 1), alongside ROS (Figure 5) and catalase activity (Figure 6) measurements, that treatment with Ag–C_60_–Cl NPs overwhelms the fungal defense systems, leading to cell death. A two-tailed *t* test statistical analysis was performed to assess the significance of differences between the nanoparticle treatments. All treatments were determined to have significant and highly significant differences (*p* < 0.05 and *p* < 0.005) indicating that Candida spp. respond distinctly to each treatment, as previously described.

### Nanoparticles-Fungi Interactions

To further elucidate the mechanisms of action for our metal-coordinated chloro-fullerenes NPs we examined direct ultrastructural effects on *C. albicans* and *C. auris* were examined using SEM (Figure 7). Both Candida species were pretreated with 0.5× MIC NPs or fluconazole and incubated for two to four hours before imaging. The SEM images of untreated *C. albicans* and *C. auris* cells revealed typical fungal growth behavior, substantiated by a dense network of agglomerated cells displaying a well-defined ovular morphology with smooth surfaces, numerous budding cells and an abundance of extracellular polymeric substances (EPS) surrounding the fungal cells. After Ag–C_60_–Cl NP treatment, the images revealed that both *C. albicans* and *C. auris* exhibited reduced cellular density, an absence of budding cells and EPS (Figure 7) and major morphological alterations. The surface appearance of the yeasts transitioned from smooth to rough, showing clear signs of cell wall damage, disruption and distortion. Similar morphological changes and EPS production were observed upon treating *C. albicans* with Cu–C_60_–Cl NPs. However, greater cell viability was observed in Cu–C_60_–Cl NPs treatments, which parallels the lower antifungal activity of the Cu–C_60_–Cl NPs (as shown in Table 1). Unlike Ag–C_60_–Cl NPs, Cu–C_60_–Cl NP treatment against *C. auris* showed a smooth surface like that of untreated cells. However, in comparison to the untreated cells, Cu–C_60_–Cl NP treatment revealed fewer budding cells. Likewise, C_60_–Cl NP treatment showed a similar effect as Cu–C_60_–Cl NPs, which explains the reduction in the growth curves (Figure 3). Notably, the observation of altered fungal cell morphology (donut-shape appearance) further validates cell wall disruption, an outcome while novel in this specific morphology, is consistent with previously reported AgNP-induced structural damage in *C. auris*.^40^

**Figure 7:**
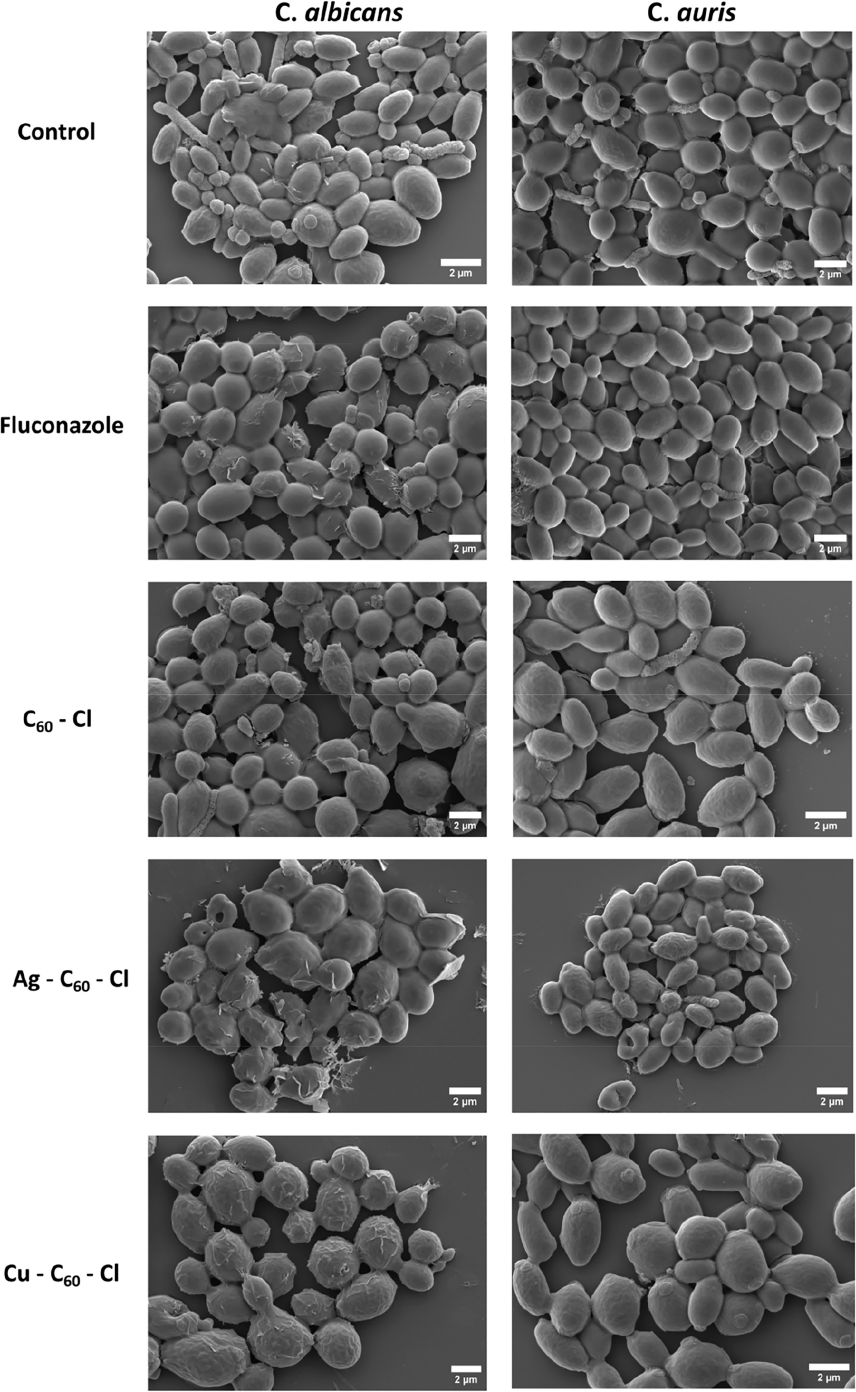
SEM images illustrating nanoparticle—fungi interactions in *C. albicans* and *C. auris* before (control) and after the treatment Ag–C_60_–Cl, Cu–C_60_–Cl, C_60_–Cl, and fluconazole. Scale bar: 2 μm.

To confirm these interactions, both fungal species were evaluated after treatment with Ag–C_60_–Cl NPs and analyzed through Scanning Electron Microscopy-Energy Dispersive X-ray (SEM-EDX) as shown in Figure 8. The elemental maps show that the Ag–C_60_–Cl NPs have higher interactions with the *C. auris* yeast cells (Figure 8B) compared to *C. albicans* (Figure 8A), indicating more pronounced NP adherence or interaction with *C. auris*. This observation aligns with the higher antifungal activity of these NPs against *C. auris* and suggests that the Ag–C_60_–Cl NPs disrupt the membrane by directly interacting with the lipid bilayers resulting in increasing the membrane permeability and facilitating the NPs penetration.^41^ As previously shown, the Ag–C_60_–Cl NPs were able to generate intracellular ROS, which resulted in oxidative stress that damages proteins, lipids, and nucleic acids.^42^ Consequently, this oxidative stress triggers cellular defense mechanisms such as catalase activity, where the cells attempt to detoxify the induced ROS damage.^42,43^ As a result of this overwhelming oxidative burden, irreversible damage occurs, leading to fungal cell death.^44^ These results support previous literature, whereby oxidative damage affects the microorganisms and compromises the structural integrity of the extracellular matrix, which further enhances the vulnerability of the biofilm to antimicrobial agents.^41^ The resultant compromised biofilm structure leads to increased porosity, which facilitates NP penetration and enhances antifungal efficacy.^45^ This dual action (i.e., the disruption of the matrix and oxidative damage to microbial cells) suggests that the Ag–C_60_–Cl NPs are highly effective against Candida spp., which corroborates the previously reported characteristics of metallic NPs.^46^

**Figure 8:**
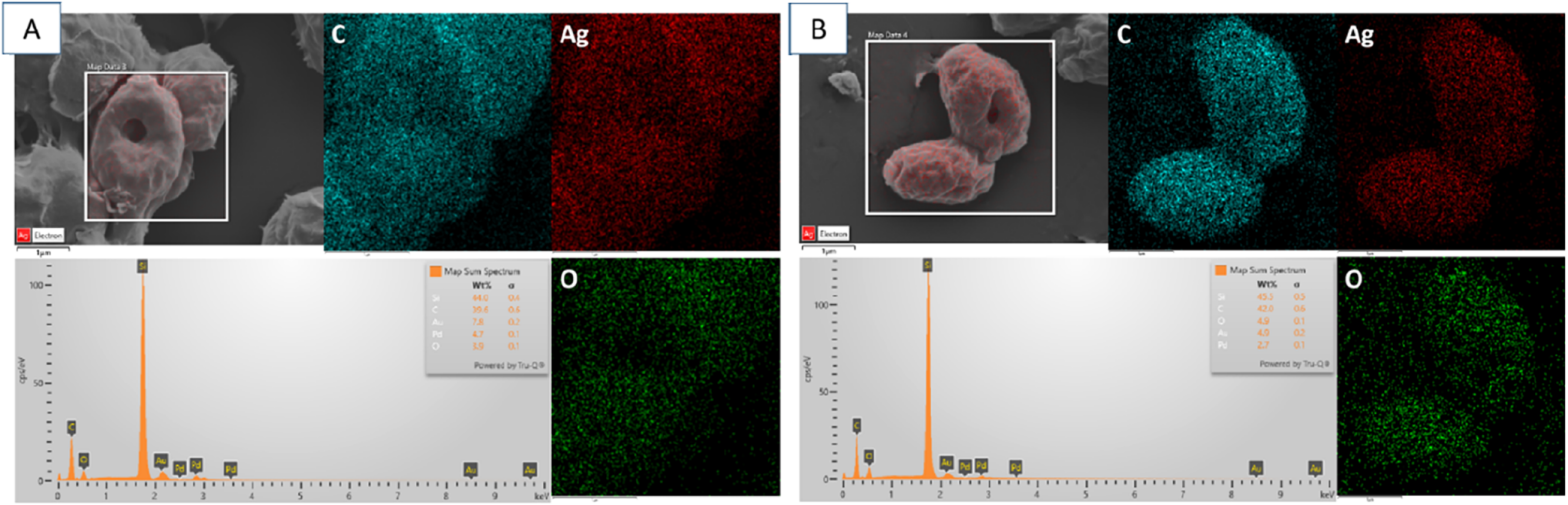
SEM-EDS analysis of the donut-shaped cells in (A) *C. albicans* and (B) *C. auris*.

### Cytotoxicity Study of the Nanoparticles

The biocompatibility of the nanoparticles was evaluated to determine cytotoxicity. The potential hemolytic effects of the NPs and fluconazole were evaluated against an untreated control specimen (PBS) and positive control (water; 100% lysis) in fresh venipuncture human whole blood (Figure 9). Increasing concentrations of the NPs (2.5, 25, 50, and 100 μg/mL) were used in addition to positive and negative controls. The results revealed hemolytic percentages below 5% for all NPs at all evaluated concentrations. Both C_60_–Cl and Cu–C_60_–Cl at MIC values (250 μg/mL) were evaluated and revealed hemolytic percentages below 5% (Data not shown). These results suggest the nonhemolytic behavior of the NPs which can be further indicated as blood-biocompatible nanoparticles according to ISO/TR 7406.

**Figure 9:**
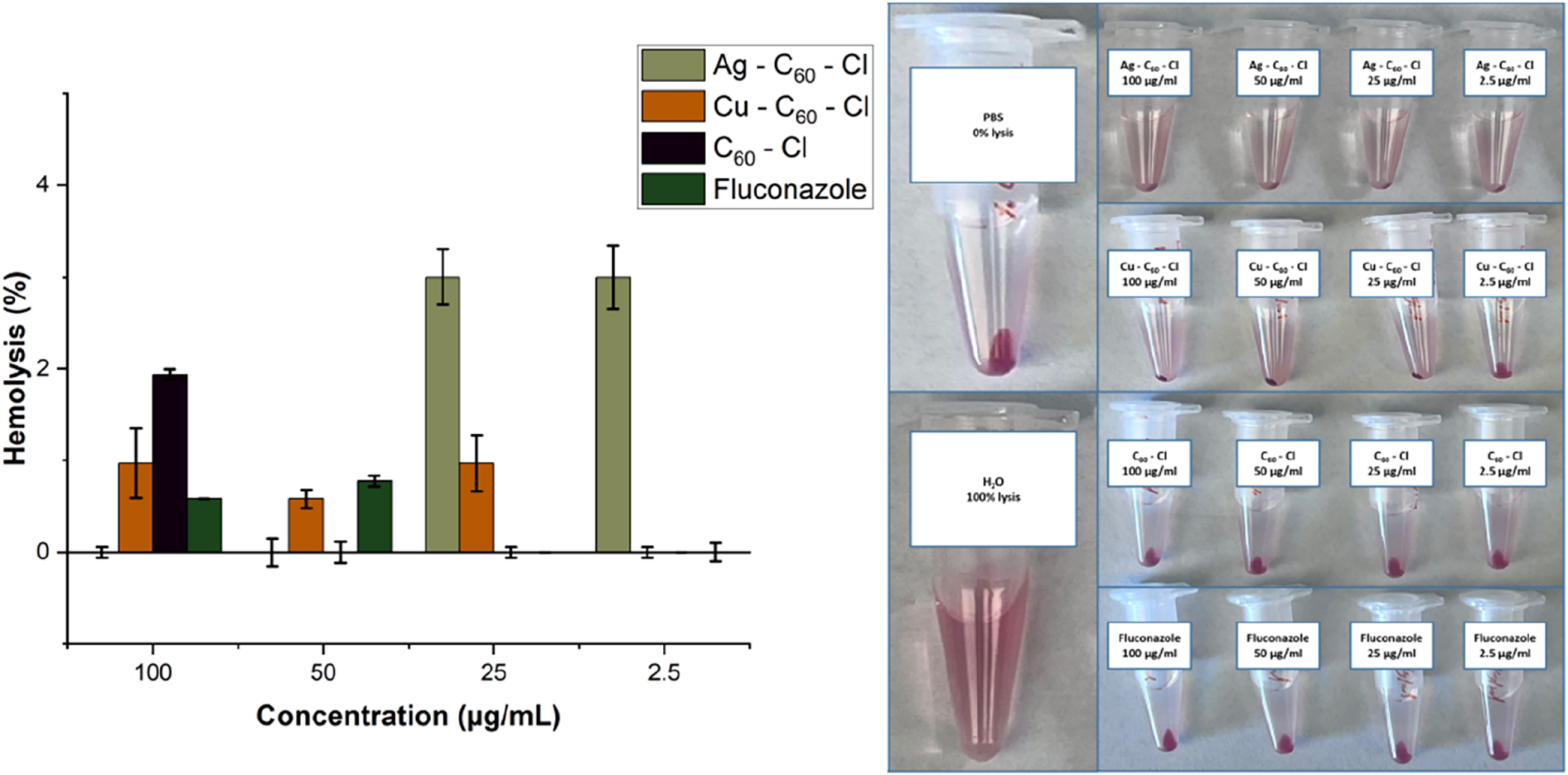
Hemolysis assay of fluconazole, C_60_–Cl, Ag–C_60_–Cl, and Cu–C_60_–Cl nanoparticles at 100, 50, 25, and 2.5 μg/mL concentrations. The hemoglobin absorbance values were measured at 540 nm. The data represent the mean values of triplicates ± SD.

## Discussion

In this study, we evaluated silver- and copper-coordinated chlorine functionalized NPs as potential fungicidal agents against *C. auris* and *C. albicans*, two clinically significant fungal pathogens with rising drug resistance concerns. To benchmark their efficacy, a nonmetallic functionalized fullerene (C_60_–Cl) and the standard-of-care azole therapy (fluconazole) were used to compare the efficacy of the synthesized NPs. The results show the potential antifungal efficacy of metallic functionalized fullerenes against *C. albicans* and *C. auris*, representing a novel fungicidal with novel mechanisms. Notably, these NPs have previously demonstrated efficacy against Gram-negative bacteria strains, including *E. coli* and MRSA,^28^ positioning them as versatile broad-spectrum antimicrobials.

*Candida auris* infections are cryptic and difficult to diagnose using conventional methods^47^ and are often associated with untimely appropriate and effective treatment (days to weeks).^48^ These challenges contribute to the high patient mortality rate associated with these infections (30–50%).^49^ In one retrospective cohort study of patients with *Candida spp*. bloodstream infections (BSI), it was found that initiating appropriate antifungal therapy within 12 h resulted in an 11.1% mortality rate, whereas delaying treatment beyond 12 h nearly tripled mortality (33.1%).^50^ Given that the diagnosis of BSIs, especially those caused by fungal pathogens, is slow and error prone (50%),^51^ clinicians must rely on broad-spectrum therapeutics and empirical treatment strategies. However, broad-spectrum antibiotics are ineffective against fungal agents, which causes further delays in the initiation of appropriate therapy. This problem is compounded by the emergence of multidrug-resistant (MDR) strains. Notably, over 90% of *C. auris* isolates are resistant to the front-line therapy, fluconazole according to the CDC.^23^ While the current antifungal armamentarium is severely limited and future solutions remain substantially lacking, these factors coalesce to describe an urgent medical need for safe, effective and innovative antifungal therapies.

Unlike *C. auris*, which is nearly entirely resistant to the standard of care treatment (fluconazole), emerging clinical evidence indicates that *C. albicans* are increasingly resistant to azole treatment, particularly in patients subjected to recurrent or prolonged therapy. While *C. albicans* is traditionally more susceptible to fluconazole than non-*albicans* fungal species (i.e., *C. glabrata* or *C. auris*), this rise in resistance mechanisms has been attributed to mutations in the ERG11 gene and the upregulation of efflux pumps. In previous studies by Pfaller and Diekema^52^ and reviews by Arendrup and Patterson,^53^ a concerning trajectory in azole resistance among clinical isolates of *C. albicans* has been well described. This trend is further supported by a large longitudinal study of *Candida* vaginitis, which reported an increase in fluconazole-resistant isolates from an average of 19% (2012–2016) to 32% (2020–2021).^54^ Additionally, recent observational studies have also identified fluconazole resistance in up to 8% of *C. albicans* isolates from urine samples.^55^ These findings serve as a clinical warning that alternative antifungal strategies are urgently needed, and those capable of circumventing established resistance mechanisms hold significant therapeutic promise.

Several studies have evaluated the antifungal activity of different types of NPs against *Candida spp*., including *C. albicans* and *C. auris*.^56,57,40^ Particularly, AgNPs have shown potent fungicidal activity through the suppression of biofilms. In Vazquez-Munoz et al., the antifungal activity of AgNPs was evaluated against *C. auris* in both planktonic and sessile biofilm.^58^ The results revealed potent antifungal activity against different strains of *C. auris* regardless their clade. According to these findings, the AgNPs prevented the formation and affected the structure of biofilms. Although AgNPs have used in the healthcare and cosmetic fields,^59^ the potential cytotoxicity risk of these NPs have restricted their systemic application in humans, which has been attributed to the complicated interactions of the living cells. In efforts to reduce the cytotoxicity of AgNPs, several research studies have evaluated green synthesized AgNPs using plant extracts.^60,61^ Moreover, some studies have introduced other metals such as copper and cobalt to mitigate any cytotoxicity concerns. For instance, Kamli et al. used a green method to synthesize Ag–Cu–Co trimetallic NPs using leave extract from *Salvia officinalis*.^62^ Their findings showed a stronger antimicrobial activity due to the synergistic effect of the three metals present. Interestingly, these results revealed no toxicity at concentrations 4-fold higher than the MIC. While many research publications have investigated the fungicidal activity of different types of NPs, many have not yet fully characterized the pharmacokinetics, pharmacodynamics, physicochemical interactions, toxicity profiles or specific mechanisms of action.^56^ This work has provided preliminary growth kinetics, interaction data and early cytotoxicity evidence to support further investigation of the proposed antifungal NP platform. Moreover, Table 3 provides a detailed comparison of the current study with the literature.

**Table 3:** Comparison of This Work with Relevant Studies from the Literature

nanomaterial	targeted fungi species	efficacy	mechanism	cytotoxicity/biocompatibility	refs
ZnO NPs	*C. albicans*	MIC = 80 μg/mL; EC_50_ = 35.6 μg/mL	dose-dependent growth inhibition; accumulation on cell surface, morphological damage via SEM, FTIR and EDX confirming interactions	not reported	^63^
Ag colloidal NPs	*C. albicans*, *C. glabrata* biofilms	biofilm study (MIC not reported)	altered biofilm matrix composition/structure; antibiofilm activity on mature biofilms.	not reported	^26^
AgNPs	*C. auris* biofilms on medical and environmental surfaces	dressings with 0.036 ppm AgNPs: >80% inhibition after washes 1–3 cycles; >50% after washes 4–6 cycles	robust, dose-dependent inhibition of *C. auris* biofilm formation on silicone and bandage fibers	authors note potential cytotoxicity of AgNPs; *in vivo* safety not assessed	^40^
biogenic AgNPs + fluconazole (nanofungicidal system)	MDR *Candida* planktonic cells and biofilms	combination enhanced elimination of MDR biofilms	reduced virulence factors (e.g., EPS/enzymes); synergistic effect with azole	biogenic synthesis reported to reduce cytotoxicity; detailed assays not reported	^21^
Ag–Ni bimetallic NPs	FLZ-resistant *C. albicans* (strain 5112)	antibiofilm at 3.12 μg/mL; hyphae inhibited at 0.78–1.56 μg/mL; efflux inhibition at 1.56 μg/mL	blocks MDR efflux pumps (R6G assays), disrupts membrane (PI uptake), alters biofilm architecture; synergy with FLZ investigated	Candida viability assay: ∼51.6% cell death at 0.5× MIC (0.78 μg/mL), ∼71.9% at MIC (1.56 μg/mL); no mammalian-cell toxicity data reported	^64^
Ag–Cu–Co trimetallic NPs (green-synthesized)	*C. auris* clinical isolates	MIC 0.39–0.78 μg/mL; MFC 0.78–1.56 μg/mL	induces apoptosis and G2/M cell-cycle arrest; enhanced activity vs monometallic counterparts	reported no toxicity up to 4× MIC (plant-mediated green synthesis)	^62^
Ag–C_60_–Cl NPs	*C. albicans* (ATCC 90028) *C. auris* (CDC PR#0385)	MIC = 15.6 μg/mL (*C. albicans*); 3.9 μg/mL (*C. auris*)	dual mechanism: membrane disruption + ROS induction; catalase activity triggered; SEM/EDS shows strong NP–cell wall interactions; significant growth inhibition	hemolysis assay showed <5% RBC lysis at concentrations up to 100 μg/mL (≈6× MIC for *C. albicans*, 25× MIC for *C. auris*)	this study
Cu–C_60_–Cl NPs	*C. albicans* (ATCC 90028) *C. auris* (CDC PR#0385)	MIC = 250 μg/mL (both species)	limited efficacy; ROS partly quenched by copper scavenging; catalase activity elevated; fungal copper homeostasis mitigates toxicity	hemolysis <5% at MIC (250 μg/mL) → nonhemolytic but low antifungal potency	this study

## Conclusions

In this study, an engineered fullerene, Ag–C_60_–Cl, showed promising early results as a novel antifungal approach that may help mitigate many current clinical challenges. These findings warrant further comprehensive safety and pharmacokinetic evaluations, as early data indicates that Ag–C_60_–Cl NPs may offer a foundation for future therapeutic development to offset delayed diagnostic turnaround times and the high mortality rates associated with fungal BSIs. In our previous research, Ag–C_60_–Cl NPs exhibited broad-spectrum efficacy, effective against Gram-negative species such as *E. coli* and WHO-priority resistant pathogens like MRSA.^28^ Herein, we extend this NP’s applicability to fungi, demonstrating potent antifungal activity at low MICs (*C. auris* = 3.9 μg/mL and *C. albicans* = 15.62 μg/mL) without hemolytic cytotoxicity, along with significant fungal growth inhibition, marked morphological disruption and robust ROS-mediated oxidative damage. While fluconazole revealed a lower MIC against *C. albicans*, its mechanism, targeting ergosterol biosynthesis, renders it less effective against resistant strains such as *C. auris*, where MIC values exceed 250 μg/mL. In contrast, the dual-action mechanism of Ag–C_60_–Cl NPs, which combines membrane disruption with oxidative stress induction, effectively circumvents conventional resistance pathways.

Mechanistic evaluations via SEM–EDX, ROS assays, and catalase activity measurements confirmed that Ag–C_60_–Cl NPs impart structural damage to the fungal cell wall and extracellular matrix while triggering oxidative stress leading to cell death. The other evaluated NP candidate, Cu–C_60_–Cl, a copper-based nanoparticles displayed reduced fungicidal activity, which is consistent with the well-documented copper-detoxification and homeostasis mechanisms in *Candida* species. While limited in scope, initial cytotoxicity assays with Ag–C_60_–Cl revealed minimal hemolytic activity at 100 μg/mL, nearly 25 times greater than the observed MIC in *C. auris* and six times higher MIC in *C. albicans*. This early safety indication shows favorable therapeutic potential, supporting Ag–C_60_–Cl as an effective fungicidal agent warranting further biocompatibility evaluations.

Overall, this work represents a possible paradigm in antifungal therapy, positioning Ag–C_60_–Cl NPs as a compelling candidate for clinical development against broad-spectrum and drug-resistant pathogens. Ag–C_60_–Cl NPs displayed greater antifungal performance, evidenced by lower MIC values, significant fungal growth inhibition, morphological disruption and ROS-related oxidative damage, when compared to control NPs (C_60_–Cl), Cu–C_60_–Cl and fluconazole. Given the global burden of MDR fungal infections, limitations of current and future antifungal agents and unfavorable diagnostic delays, these findings show the translational promise of strategically functionalized and coordinated metallofullerene NPs. The unique physiochemical properties of fullerenes, functional capabilities, microbiocidal activity and safety profiles warrant further investigation as systemic antimicrobial agents administered orally or parentally, as topical antimicrobial agents or as antifungal coatings for indwelling catheters and other medical devices and surfaces to provide a multifaceted approach to overcoming resistance. Future *in vivo* studies to validate biocompatibility, pharmacokinetics and broad spectrum, synergistic effects with existing therapies and expanded fungi evaluation are warranted.

## Supplementary Material



## Notes

C_60_–Cl; SEM image of (a) C_60_–Cl, (b) Ag–C_60_–Cl, and (c) Cu–C_60_–Cl NPs, and characterization data for the NPs (PDF)
