# Supplementary material for: Impact of Metal-Functionalized Fullerenes on the Proliferation of Pathogenic Fungi
Source: ACS Omega. 2026 Feb 2;11(7):11168–82. doi: 10.1021/acsomega.5c07052 (PMC12947005; doi:10.1021/acsomega.5c07052)
Supplement: Supplementary file 1 [file ao5c07052_si_001.pdf]

SUPPORTING INFORMATION

Impact of Metal-Functionalized Fullerenes on the Proliferation of Pathogenic Fungi

Abed Alqader Ibrahim[1], Tariq Khan[1], Dennis LaJeunesse[1], Sherine O. Obare[1*], Anthony L. Dellinger[1,2,3*]

[1]Department of Nanoscience, Joint School of Nanoscience and Nanoengineering, University of North Carolina at Greensboro, Greensboro, NC, 27401, United States. (amibrahim@uncg.edu), (t_khan4@uncg.edu), (drlajeun@uncg.edu), (soobare@uncg.edu), and (aldellin@uncg.edu) [2]Kepley Biosystems Incorporated, Greensboro, NC, 27214, United States. [3] AT Research Partners, Burlington, NC, 27217, United States.

Dynamic Light Scattering (DLS)

Table S1: **C_{60}-Cl**

Sample Name:	Measurement Start Date and Time				
C60-Cl	10/10/2024 11:29				
C60-Cl	10/10/2024 11:30				
Name	Mean	Standard Deviation	RSD	Minimum	Maximum
Z-Average (nm)	726.875	68.588	9.436	678.376	775.375
Polydispersity Index (PI)	0.464	0.2	43.209	0.322	0.605
Peak 1 Mean by Intensity ordered by area (nm)	526.69	73.891	14.029	474.441	578.939
Peak 1 Area by Intensity ordered by area (%)	99.157	1.191	1.202	98.315	100
Peak 2 Mean by Intensity ordered by area (nm)	5467.693	0	0	5467.693	5467.693
Peak 2 Area by Intensity ordered by area (%)	1.685	0	0	1.685	1.685

Table S2: **Ag-C_{60}-Cl**

Sample Name:	Measurement Start Date and Time				
Ag-C60-Cl	10/10/2024 10:57				
Ag-C60-Cl	10/10/2024 10:58				
Ag-C60-Cl	10/10/2024 10:59				
Name	Mean	Standard Deviation	RSD	Minimum	Maximum
Z-Average (nm)	827.305	63.316	7.653	754.197	864.344
Polydispersity Index (PI)	0.572	0.037	6.493	0.54	0.613
Peak 1 Mean by Intensity ordered by area (nm)	443.916	43.485	9.796	409.631	492.83
Peak 1 Area by Intensity ordered by area (%)	100	0	0	100	100

Table S3: **Cu-C_{60}-Cl**

Sample Name:	Measurement Start Date and Time				
Cu-C60-Cl	10/10/2024 11:50				
Cu-C60-Cl	10/10/2024 11:51				
Cu-C60-Cl	10/10/2024 11:52				
Name	Mean	Standard Deviation	RSD	Minimum	Maximum
Z-Average (nm)	1044.481	56.993	5.457	984.882	1098.45
Polydispersity Index (PI)	0.941	0.054	5.784	0.893	1
Peak 1 Mean by Intensity ordered by area (nm)	436.625	41.361	9.473	410.955	484.339
Peak 1 Area by Intensity ordered by area (%)	98.307	2.932	2.983	94.921	100
Peak 2 Mean by Intensity ordered by area (nm)	111.778	0	0	111.778	111.778
Peak 2 Area by Intensity ordered by area (%)	5.079	0	0	5.079	5.079

Zeta potential:

Table S4: **C_{60}-Cl**

Sample Name:	Measurement Start Date and Time				
C60-Cl	10/7/2024 13:18				
C60-Cl	10/7/2024 13:20				
C60-Cl	10/7/2024 13:22				
Name	Mean	Standard Deviation	RSD	Minimum	Maximum
Zeta Potential (mV)	-58.375	0.692	1.186	-59.124	-57.759
Conductivity (mS/cm)	0.005	0	0	0.005	0.005
Wall Zeta Potential (mV)	-81.219	2.188	2.694	-83.669	-79.459
Quality Factor	6.968	1.285	18.449	6.195	8.452
Zeta Peak 1 Mean (mV)	-58.413	0.642	1.1	-59.124	-57.874
Zeta Peak 2 Mean (mV)	-33.899	0	0	-33.899	-33.899

Table S5: **Ag-C_{60}-Cl**

Sample Name:	Measurement Start Date and Time				
Ag-C60-Cl	10/7/2024 13:45				
Ag-C60-Cl	10/7/2024 13:46				
Ag-C60-Cl	10/7/2024 13:48				
Name	Mean	Standard Deviation	RSD	Minimum	Maximum
Zeta Potential (mV)	-47.48	2.746	5.784	-49.317	-44.323
Conductivity (mS/cm)	0.008	0	0	0.008	0.008
Wall Zeta Potential (mV)	-59.093	4.8	8.123	-64.455	-55.197
Quality Factor	3.593	0.778	21.648	3.081	4.488
Zeta Peak 1 Mean (mV)	-47.48	2.746	5.784	-49.317	-44.323

Table S6: **Cu-C_{60}-Cl**

Sample Name:	Measurement Start Date and Time				
Cu-C60-Cl	10/7/2024 13:27				
Cu-C60-Cl	10/7/2024 13:29				
Cu-C60-Cl	10/7/2024 13:31				
Name	Mean	Standard Deviation	RSD	Minimum	Maximum
Zeta Potential (mV)	25.402	1.292	5.086	24.565	26.89
Conductivity (mS/cm)	0.543	0	0	0.543	0.543
Wall Zeta Potential (mV)	-8.674	1.361	15.687	-10.111	-7.405
Quality Factor	2.655	1.025	38.593	1.727	3.754
Zeta Peak 1 Mean (mV)	25.402	1.292	5.086	24.565	26.89

Scanning Electron Microscopy (SEM)

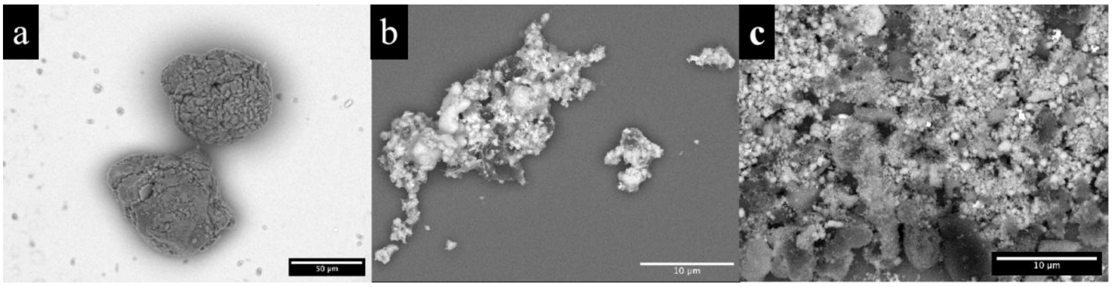

Figure S1: SEM image of (a) C_{60}-Cl, (b) Ag-C_{60}-Cl, and (c) Cu-C_{60}-Cl NPs.

Energy Dispersive X-ray Spectrometer (EDS)

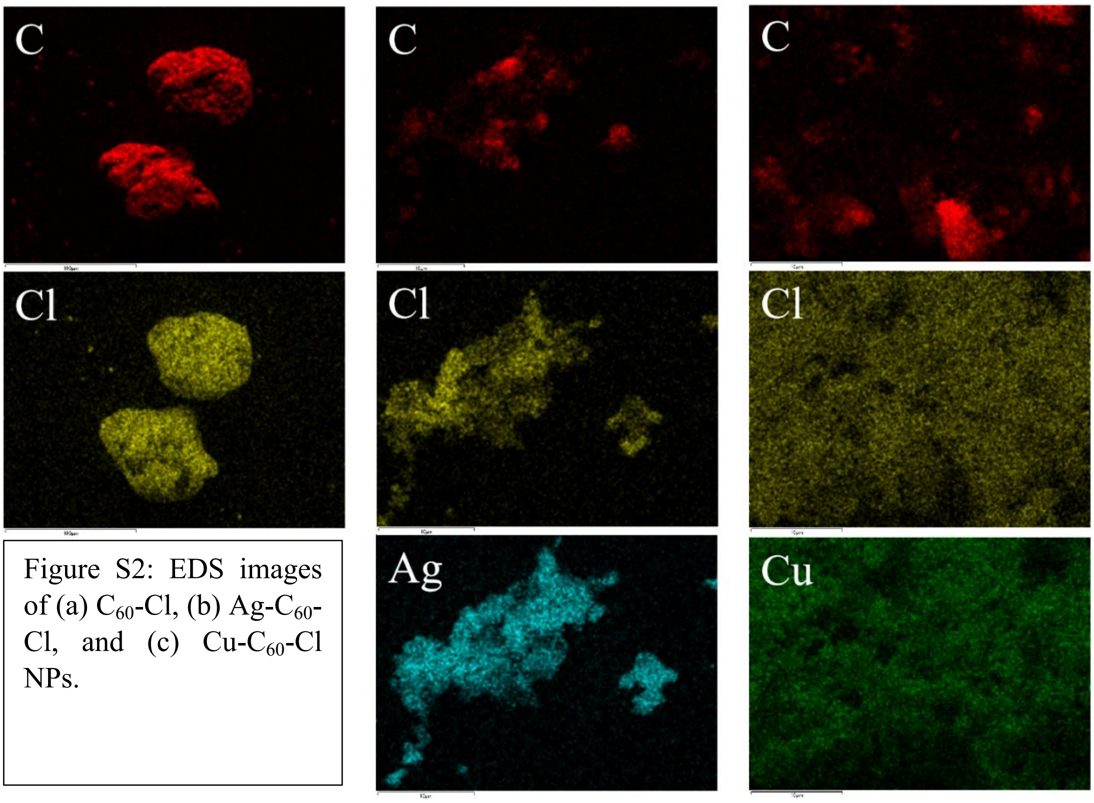

Figure S2: EDS images of (a) C_{60}-Cl, (b) Ag-C_{60}-Cl, and (c) Cu-C_{60}-Cl NPs.

Transmission Electron Microscopy (TEM)

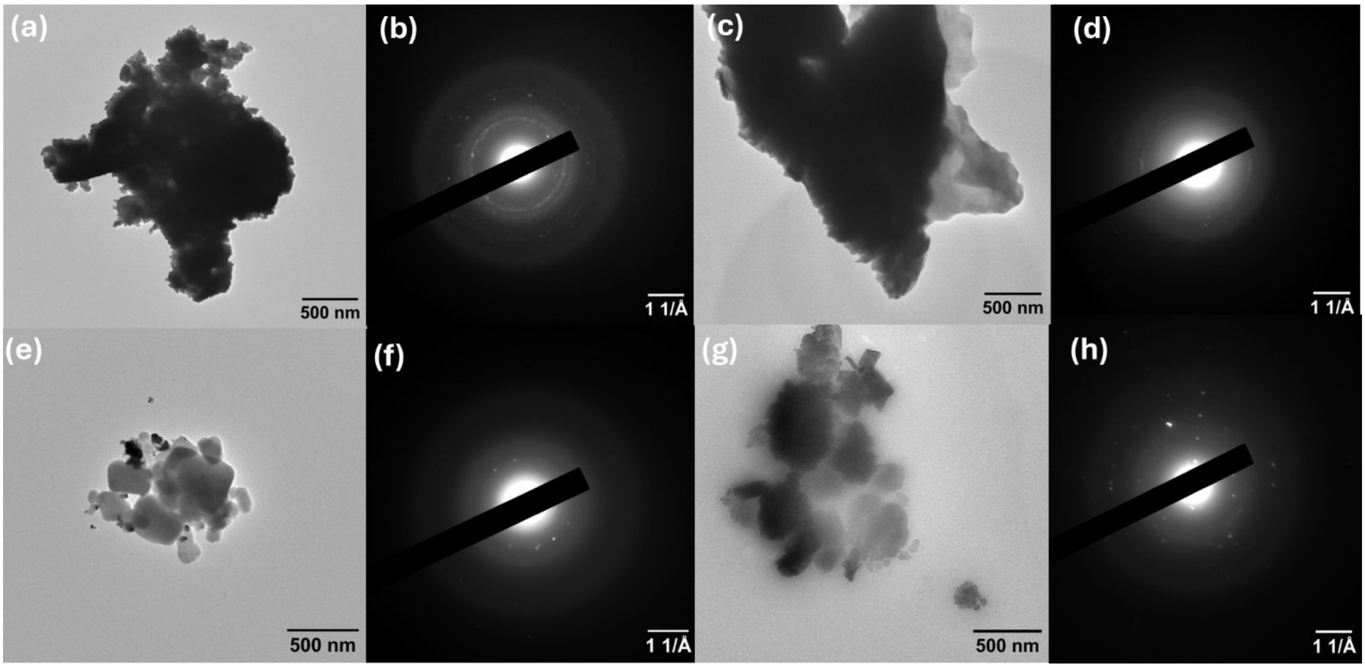

Figure S3: TEM images and corresponding SAED pattern of (a), (b) C_{60} NPs at 10k×; (c), (d) C_{60}-Cl NPs at 10k×; (e), (f) Ag-C_{60}-Cl NPs at 10k×; (g) and (h) Cu-C_{60}-Cl at 10k×.
